# Direct Fabrication of Micron-Thickness PVA-CNT Patterned Films by Integrating Micro-Pen Writing of PVA Films and Drop-on-Demand Printing of CNT Micropatterns

**DOI:** 10.3390/nano11092335

**Published:** 2021-09-08

**Authors:** Jun Luo, Zhixuan Zhao, Lehua Qi, Hongcheng Lian, Yufang Zhao

**Affiliations:** 1 School of Mechanical Engineering, Northwestern Polytechnical University, Xi’an 710072, China; zhaozhixuan97@mail.nwpu.edu.cn (Z.Z.); 2015100416@mail.nwpu.edu.cn (H.L.); 2020100897@mail.nwpu.edu.cn (Y.Z.); 2 Research & Development Institute of Northwestern Polytechnical University in Shenzhen, School of Mechanical Engineering, Northwestern Polytechnical University, Xi’an 710072, China

**Keywords:** hybrid printing, micro-pen writing, drop-on-demand printing, droplet spreading, thin viscoelastic substrate, multi-layer overwriting

## Abstract

The direct fabrication of micron-thickness patterned electronics consisting of patterned PVA films and CNT micropatterns still faces considerable challenges. Here, we demonstrated the integrated fabrication of PVA films of micron-thickness and CNT-based patterns by utilising micro-pen writing and drop-on-demand printing in sequence. Patterned PVA films of 1–5 μm in thickness were written first using proper micro-pen writing parameters, including the writing gap, the substrate moving velocity, and the working pressure. Then, CNT droplets were printed on PVA films that were cured at 55–65 °C for 3–15 min, resulting in neat CNT patterns. In addition, an inertia-pseudopartial wetting spreading model was established to release the dynamics of the droplet spreading process over thin viscoelastic films. Uniform and dense CNT lines with a porosity of 2.2% were printed on PVA substrates that were preprocessed at 55 °C for 9 min using a staggered overwriting method with the proper number of layers. Finally, we demonstrated the feasibility of this hybrid printing method by printing a patterned PVA-CNT film and a micro-ribbon. This study provides a valid method for directly fabricating micron-thickness PVA-CNT electronics. The proposed method can also provide guidance on the direct writing of other high-molecular polymer materials and printing inks of other nanosuspensions.

## 1. Introduction

Since carbon nanotubes (CNTs) have excellent properties, such as high strong mechanical stability, electrical conductivity, and good compatibility with polymer films, micron-thickness CNT-based electronics consisting of polymer films and CNT micropatterns have wide applications in supercapacitors [1,2], sensors [3,4,5,6], solar cells [7], and other functional devices [8,9,10]. With the growing demand for device performance enhancement, the design of polymer substrates and CNT micropatterns has become more complex [11,12] and tends to be multi-layered [13,14]. Hereby, there is a solid demand for the direct manufacturing of patterned micron-thickness CNT electronics, including micron-thickness polymer substrates and CNT micropatterns.

Unfortunately, the fabrication of custom-patterned polymer films of micron thickness with a smooth surface is still very challenging. Spin coating [15,16], solution casting [17,18], and vapour solvent deposition method [19,20] are widely used for preparing polymer films of electronics. Compared with these technologies, the micro-pen writing method can directly prepare multi-layer patterned films of polymer of micron thickness without using high-temperature and energy-intensive processes. In addition, the micro-pen writing method is the most suitable for direct writing high viscosity hydrogels. The drop-on-demand (DoD) printing technology [21,22,23,24] provides a low-cost, high-precision method for printing conductive micropatterns on written polymer substrates. However, the wetting effect of nanosuspension droplets spreading over substrates [25,26,27] is hard to control, and the coffee-ring phenomenon [28] is caused by uneven evaporation. These mechanisms reduce the uniformity and density of printed conductive patterns, limiting the enhancement of properties of the printed devices.

Many efforts are still being made to enhance the uniformity and density of printed micropatterns. Shin [29] enhanced the surface energy of PET films as the substrate of printing to match the surface tension of graphene oxide (GO) ink to inhibit the spreading of a single GO ink droplet. Uniform GO lines were printed on the treated film. Lian [30] printed uniform nitrogen-doped graphene (NG) lines with favourable outlines on the glass slide by regulating the temperature of the substrate and the spacing. The density of the NG lines was improved by overwriting proper layers. In their work, the deformation of the substrate caused by the impact and spreading of ink droplets could be ignored since the substrate surface was rigid. However, the deformation of the substrate caused by the droplet impacting the substrate and spreading could significantly affect the morphology of the solute produced upon evaporation of the liquid ink.

To further study the morphology of ink solute on a deformable substrate, Chen [31] demonstrated that the viscoelasticity of substrates could affect the evaporation of sessile nanosuspension droplets (0.125 wt.% SiO_2_–water solution) on viscoelastic substrates (polydimethylsiloxane (PDMS)), resulting in different deposition morphologies. Iqbal [32] reduced the diameters of deposition patterns of suspension droplets (polystyrene microparticles + DI water) by decreasing the elastic modulus of the substrate (Sylgard 184), improving the resolution of a single-droplet deposition on elastic substrates. Nevertheless, printing patterns of nanoparticles on these elastic/viscoelastic substrates was not carried out. Besides, these investigations did not take into account the effect of the rigid support substrate on the droplet dynamic behaviours on the deformable substrate.

It is important to note that a rigid support can significantly affect the dynamics of the droplet on the viscoelastic substrate when the thickness is small enough compared with the diameter of the droplet [33]. However, studies on the nanosuspension droplet deposition morphology still focused on rigid or thick viscoelastic substrates. Different from these substrates, the thin viscoelastic substrate influences the droplet spreading by generating a ridge. Furthermore, the deposition process of droplets is affected by the rigid surface that supports the thin film. There is little systematic research on the deposition and spreading behaviour of nanosuspension droplets on the thin viscoelastic film and the regulation of deposition morphology, which is necessary for nanosuspension printing on a thin viscoelastic substrate.

In the present paper, we introduce a novel way to fabricate patterned micron-thickness carbon-based electronics. To this end, thin PVA films of micron thickness were directly written using a micro-pen and then CNT suspension droplets were printed on-demand on the PVA films to form patterns. The effects of the writing gap, the writing velocity, and the working pressure on the thickness of the written substrate were systematically investigated. The written substrates were heated to different temperatures for different durations. The spreading behaviours of a single sessile CNT droplet on PVA substrates under different preprocessed conditions were observed and analyzed. The resolution of deposition morphologies was compared to each other for choosing the optimal substrate condition. Finally, uniform and dense straight lines of CNTs were printed on the best preprocessed substrate using a staggered printing strategy and overwriting the proper number of layers.

## 2. Materials and Methods

### 2.1. Solution

A self-developed 6%PVA-10% H_2_SO_4_ hydrogel was used to write PVA films via micro-pen writing. The density of this PVA hydrogel was 1.02 g/mL. A PVA film with good flexibility can be obtained by writing this hydrogel without high-temperature treatment procedures.

The ink used for the DoD printing micropatterns was the isopropanol CNT suspension that was diluted from the CNT suspension that was produced by the TIMENANO company (Chengdu, China). The IPA-CNT ink obtained favourable dispersion stability after it was treated ultrasonically for 20 min at 25 °C. The CNT ink exhibited good dispersibility without precipitation. At a concentration of 2 mg/mL, the CNT ink had a surface tension of 22 mN/m at 25 °C and a density of 0.82 g/mL.

### 2.2. Hybrid Printing Setup and Procedure

The experiments were carried out on proprietary hybrid printing equipment combined with a micro-pen writing system and droplet-printing equipment (see Supplementary Materials). A 3D nano-piezo stage (M410.DG, Pi, Karsruhe, Germany) was used to coordinate the film writing and the CNT micropatterns printing.

**Figure 1:**
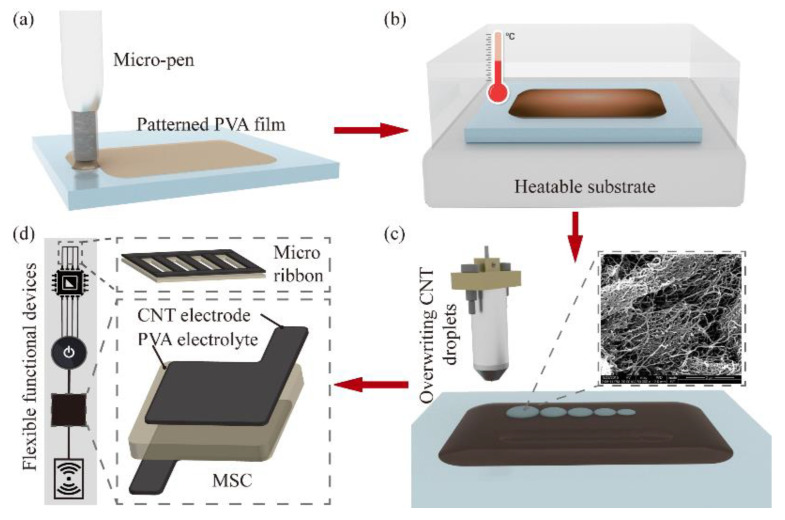
Schematic illustration of hybrid printing method for PVA-CNT flexible electronics fabrication. (**a**) Micro-pen writing was used to pattern the PVA film on the slide substrate. (**b**) Preprocessing of the written PVA film using heating in place. (**c**) CNT droplet DoD printing on the processed patterned PVA film. (**d**) Printed flexible PVA-CNT micro-ribbon circuit and the flexible patterned PVA-CNT film as an essential element of the MSC.

The micro-pen writing system was composed of a picolitre pump (LPP01-100, LONGER, Baoding, China) and a glass capillary nozzle with an inner diameter of 750 μm. The nozzle was used to inhale the PVA hydrogel and form a liquid bridge, as shown in Figure 1a.

Prior to the PVA writing, the writing substrates (pristine glass slides) were ultrasonically treated for 25 min, rinsed with DI water, and dried. During the PVA film writing, an ultraclean slide was fastened on the heatable substrate, which was fixed on a 3D nano- piezo stage. Once the patterned PVA thin film was written, it was heated in different conditions to decrease the viscoelastic modules and change the surface energy for the deposition of the CNT droplets, as shown in Figure 1b.

A self-developed uniform nano-carbon droplet printing equipment [30,34] was used for printing the CNT micropatterns, as shown in Figure S2 (see Supplementary Materials). The CNT droplets could be stably ejected without satellite droplets (Figure 1c) with the appropriate pulse amplitude and pulse width. As shown in Figure 1d, micron-thickness flexible electronics with complex patterns, such as a micro-supercapacitor (MSC) and a micro-ribbon, can be printed using the developed hybrid printing system. All experiments were carried out in a cleanroom under an ambient temperature of 25 ± 1 °C and relative humidity of 12 ± 4%.

### 2.3. Characterisation Methods

The surface tension and dynamic viscosity of the PVA hydrogel and the CNT suspension were characterised using a surface tensiometer (A601, KINO, Boston, MA, USA) and a rotational rheometer (R/S Plus, AMETEK Brookfield, Middleboro, MA, USA), respectively. The spreading and evaporation processes of the CNT droplets on thin PVA films were recorded using a high-speed CCD camera (I-Speed 220, IX Cameras, Essex, UK) with a temporal resolution of 1000 frames per second, equipped with a monocular microscope and a cold light source, The appearance and contours of patterned PVA films were characterised by the optical profiler (NT1100, Veeco, Plainview, NY, USA). The morphologies of the printed PVA films and CNT patterns were characterised using optical microscopy (ECLIPSELV150N, Nikon, Tokyo, Japan).

## 3. Results and Discussion

### 3.1. Effect of Writing Parameters and Heat Treatment on the Patterned PVA Film

**Figure 2:**
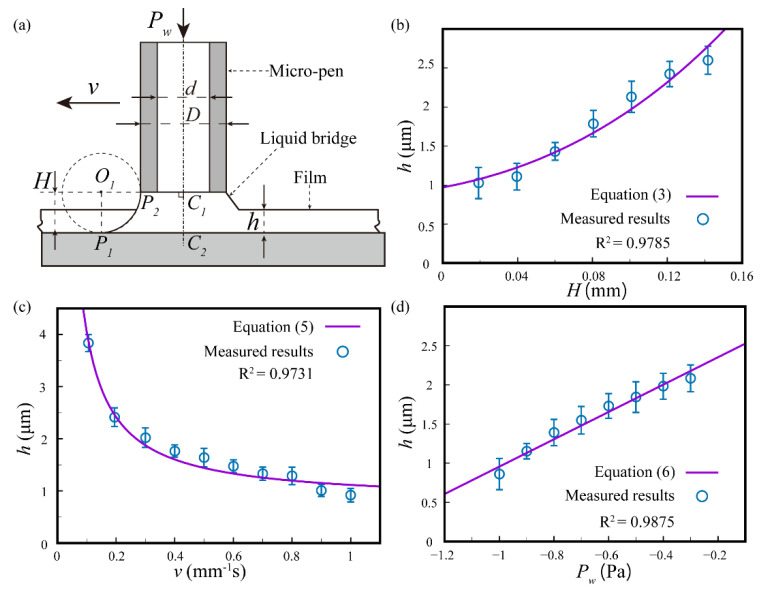
Effect of the micro-pen writing parameters on the PVA film thickness. (**a**) Schematic diagram of the micro-pen writing process. (**b**) Effect of the writing gap *H* on the film thickness. (**c**) Effect of the substrate moving velocity *v* on the film thickness. (**d**) Effect of the working pressure *P_w_* on the film thickness.

To fabricate the patterned films of micron thickness, the PVA hydrogel was extruded from the head of the nozzle while the substrate was moved along the specified path. The PVA hydrogel was stored in a chamber and was extruded using N_2_ gas onto the moving substrate, as shown in Figure S1 (see Supplementary Materials). As shown in Figure 2a, the total PVA fluid volume *V*_t_ in the writing process was composed of the residual volume of the micro-pen cavity *V*_r_, the volume of the liquid bridge *V*_LB_ between the substrate and the lower end of the micro pen, and the volume of the film *V*_f_. The appropriate PVA film thickness could be obtained by adjusting micro-pen writing parameters, including the writing gap *H*, the substrate moving velocity *v*, and the working pressure *P_w_*.

The volume of a liquid bridge *V*_LB_ can be calculated geometrically according to its shape characteristics. Figure 2b–d displays the geometric representation of the cross-section of the liquid bridge. *O*_1_ is the centre of the circle located outside of the liquid bridge. The liquid bridge can be seen as arc *P*_1_*P*_2_ rotating along axis *C*_1_*C*_2_. The coordinate system was established by taking the point *C*_1_ as the origin, the line *C*_1_*C*_2_ as the *x*-axis, and *C*_1_*O*_1_ as the *y*-axis. The equation of the arc *P*_1_*P*_2_ can be expressed as:

(1) $$x^{2}+[{\left(y-(\frac{D}{2}+H)\right]}^{2}=H^{2}$$

The liquid bridge volume *V*_LB_ is obtained by integrating the arc *P*_1_*P*_2_ around the *x*-axis:

(2) $$V_{\mathrm{LB}}=\frac{1}{12}\pi [(20+6\pi )H^{3}+3D(4+\pi )H^{2}+3D^{2}H]$$

By substituting $V_{\mathrm{LB}}=V_{f}-V_{t}-V_{r}$ into Equation (2), the approximate equation of the PVA film thickness *h* is:

(3) $$h=\frac{(10.17H^{3}+4.21H^{2}+0.44H)+\pi {\left(\frac{D}{2}\right)}^{2}\Delta L}{A},$$

where *D* is the outside diameter of the micro-pen, Δ*L* is the height difference between before and after the hydrogel writing in the glass capillary nozzle, and *A* is the surface area of the written PVA film.

During the writing process, the liquid bridge volume was assumed to remain constant. According to the principle of flow conservation, the outflow rate in the micro-pen cavity is equal to the film volume formed by writing. Because the flow velocity of the hydrogel is slow enough inside the micro-pen cavity, the hydrogel flow can be treated as a steady laminar flow. The volume flow rate *Q* of the micro-pen laminar flow is:

(4) $$Q=\frac{\pi d^{4}}{128\mu L}(\Delta P+\rho gL)=hwv,$$

where *d* is the inner diameter of the micro-pen cavity; *L* is the initial liquid height in the cavity; and *μ* and *ρ* are the dynamic viscosity and the density of PVA hydrogel, respectively. Δ*P* is the pressure difference between the working pressure *P_w_* and the atmospheric pressure, *v* is the velocity of the substrate, and *w* is the width of each writing film. When there is a constant working pressure *P_w_* and writing gap *H*, the film thickness *h* can be expressed as:

(5) $$h=\frac{\pi d^{4}}{128\mu Lwv}(\Delta P+\rho gL)$$

Equation (5) indicates that the film thickness *h* is inversely proportional to the substrate velocity *v*.

The volume of the liquid bridge can be seen as constant in the dynamic process of the film writing. When the substrate velocity and the writing gap are constant, the film thickness can be estimated by:

(6) $$h=\frac{\pi d^{4}}{128\mu Lwv}\Delta P+\frac{\pi d^{4}}{128\mu Lwv}\rho gL$$

Equation (6) shows a linear relationship between the film thickness *h* and the pressure difference Δ*P*.

Film-writing experiments were carried out to manufacture flawless patterned films with different thicknesses. A vertical piezo stage was used to control the writing gap *H*, which increased from 20 to 140 μm. The writing experiments were carried out under the substrate velocity of 0.05 mm/s and the working pressure of −0.2 MPa. Figure 2b shows the positive correlation of the writing gap *H* on the film thickness *h*. For the fixed micro-pen inner diameter of 750 μm, the gravity of the PVA hydrogel dominated the writing process rather than the surface tension and viscosity force. When *H* was increased, the PVA hydrogel accumulation increased with a constant *P* and *v*. Figure 2c shows an inverse proportional relationship between the substrate velocity *v* and the film thickness *h* under a working pressure of −0.2 MPa and a writing gap of 20 μm. Figure 2d shows a positive linear correlation between the working pressure *P_w_* and the film thickness *h* when the substrate velocity *v* was 1 mm/s and the writing gap *H* was 0.02 mm. Figure 2b–d shows good correlations between the experimental results and the theoretical curve with all R^2^ > 0.97.

**Figure 3:**
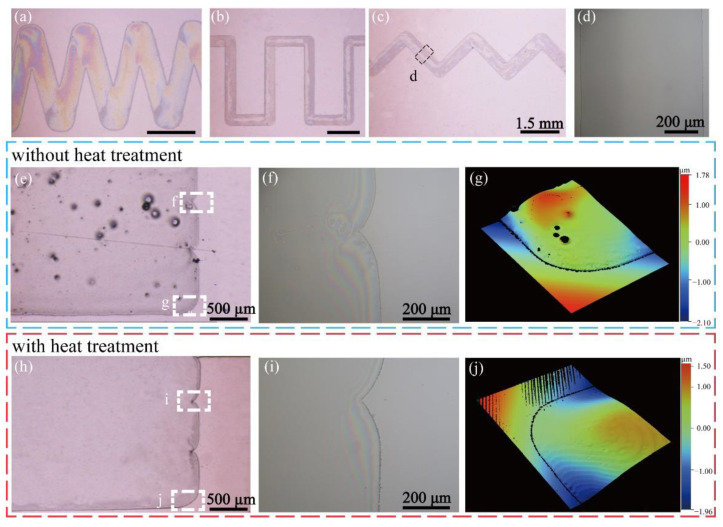
Optical microscope images of patterned PVA films that were prepared using micro-pen writing. (**a**–**c**) Different patterned PVA films. All scale bars in (**a**–**c**) are 1.5 mm. (**d**) Partial enlarged drawing of the patterned PVA film. (**e**) The written PVA film was placed in the cleanroom for 1 h without heat treatment. (**h**) The written PVA film was heated at 55 °C for 9 min after writing and placed in the cleanroom for 1 h. (**f**,**i**) Interference fringes of the combined area photographed using an optical microscope. (**g**,**j**) Fringe thickness measurement using an optical profilometer.

To demonstrate the flexibility of micro-pen writing, different patterned PVA films based on lines were written by controlling the piezo stage’s movement, as shown in Figure 3a–c. Figure 3d shows the minimum resolution of the PVA lines of 752.9 μm by using the nozzle with a diameter of 750 μm. However, film defects, including wrinkles, pores, and uneven film contours, existed due to the local cooling of its surface, as shown in Figure 3e–g. In Figure 3f, interference fringes that were generated by light on the joint of two lines extended from the edge to the centre, indicating that the combined area had slight wrinkles. In the PVA hydrogel, some of the H_2_O molecules failed to form hydrogen bonds with the hydroxyl groups of PVA molecules, which led to the evaporation of these water molecules to form pores when the film was placed at room temperature. The uneven distribution of surface tension caused by the evaporation of water on the film’s surface induced wrinkles. According to Zhou [35], the heat treatment of a PVA film can increase its fluidity by reducing its viscosity and rely on it to repair the formed pores and wrinkle defects. It is necessary to heat the written film for a moderate amount of time in a specific temperature environment. Additionally, the smooth surface of PVA films should be achieved by self-levelling after the films were written along the programmed path.

The heating test of PVA films shows that the PVA film of 5 μm thickness obtained using spin coating deteriorated at 70 °C, which was manifested as the generation of pores and the precipitation of particles. Smooth and uniform surfaces were obtained by heating at 55, 60, and 65 °C for 3–15 min. Figure 3h shows a patterned PVA film without obvious pores and wrinkles, which was obtained by treating at 55 °C for 9 min. In this case, interference fringes that were generated by light on the edge of the film were almost straight, indicating that the combined area was smooth (Figure 3i). No defects on the film fringe were observed after the heating process (Figure 3j), indicating the feasibility and the reliability of the micro-pen writing method.

Based on the above discussion, patterned PVA films could be prepared in the thickness range from 0.7 to 5 μm by adjusting the three parameters (i.e., the writing gap *H*, the substrate moving velocity *v*, and the working pressure *P_w_*). Finally, PVA films with a neat surface that was free of obvious pores and wrinkles could be obtained by heating the written film at 55–65 °C for 3–15 min. Such films were essential for the subsequent DoD printing of CNT droplets.

### 3.2. Effect of Heating Temperature and Heating Time of PVA Film on the Droplet Spreading

Different heating temperatures and heating time treatments lead to the differences in properties of PVA films. According to Shin [29] and Iqbal [32], the surface energy and the viscoelastic modulus of preprocessed PVA films can significantly influence the morphologies of deposited CNT droplets. To reveal the relationship between the surface properties (viscoelastic modulus and surface energy) of PVA films after the heating treatment and the morphologies of printed droplets, and hence choose the appropriate film for the next printing, CNT droplets were printed on PVA films that were pre-heated to different temperatures (55, 60, and 65 °C) and times (3, 6, 9, 12, and 15 min) at room temperature. In our experiments, the diameter of the droplet *d*_drop_ is 135 ± 2 μm, jetting under proper voltage and pulse length without satellite droplets, and the thickness of the PVA film *h* used in the droplet deposition experiment was 5 μm.

**Figure 4:**
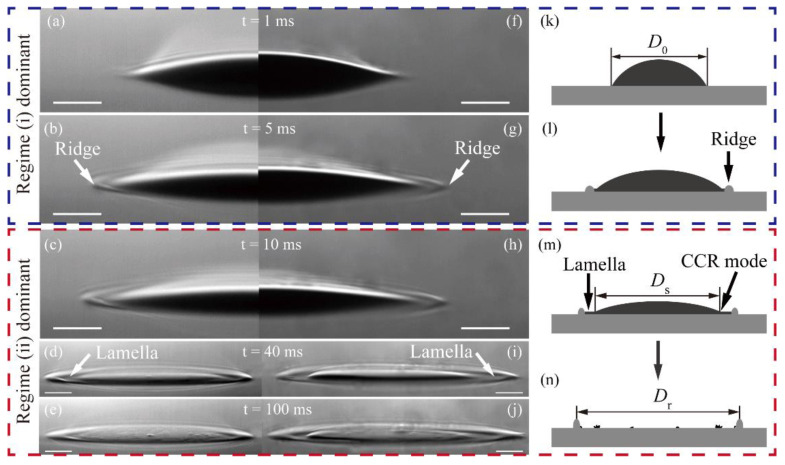
The sequence of events from a CNT droplet’s contact with different films to evaporation. ((**a**–**e**), left) The film was preprocessed at 55 °C for 9 min. ((**f**–**j**), right) The film was preprocessed at 65 °C for 9 min. (**k**–**n**) Schematic of the spreading and evaporation process of a single CNT droplet. All scale bars are 30 μm in (**a**–**j**).

Due to the influence of the rigid substrate below the thin film, the behaviour of droplets on thin viscoelastic films is significantly different from that on the thick and soft substrate [31,33] (see Appendix A). To probe the energy changes during the droplet collision and spreading processes, we analyzed the droplet behaviour using time-resolved images and research experience. As shown in Figure 4a–j, the continuous forward motion of the contact line is observed within the first five milliseconds, accompanied by the viscoelasticity ridge, which was pushed by a widening liquid rim around the contact line. Then, the spreading droplet began to display a CCR (constant contact radius) evaporation mode as soon as the contact line was pinned on the film at about 10 ms. The ridge on the 65 °C-9 min film expanded faster and broader than the one on the 55 °C-9 min film.

Based on the above and the research of Kajiya [36], the process of the droplet spreading and pushing the ridge of the viscoelastic film could be divided into two regimes. In regime (i), as shown in Figure 4a,b,k,l, the PVA film responded as an elastic solid to the motion of the contact line. In regime (ii), as shown in Figure 4c–e,m,n, the PVA film behaved nearly like a sheet of high viscosity liquid with ridge motion.

In the highly transient process of a droplet impinging a film, the flow is redirected from a vertical to a horizontal flow direction [37]. It generates outer capillary surface waves, as shown in Figure 4b,c,g,h by perturbing the film. A large part of the initial kinetic energy is consumed to create a crater with a height that is comparable to the thickness of the film and overcome the resistance caused by film deformation, which can be expressed as:

(7) $$\Delta E_{r1}=f_{1}\frac{\sigma^{2}}{2G\varepsilon }\int_{0}^{t_{i}}v(t)D(t)dt,$$

where *f*_1_ is the ratio of the imaginary and real components of the elastic modulus in the impact process; *ε* is an empirically determined distance near the triple line; *G* is the shear elastic modulus of PVA film; *t_i_* is the impacting time; and *v*(*t*) and *D*(*t*) are the velocity and diameter of the droplet during impact process, respectively [38,39,40]. Furthermore, part of the initial kinetic energy dissipates during expansion due to viscous force, which can be expressed as:

(8) $$\Delta E_{v1}=\int_{0}^{t_{i}}\int_{V}\mu_{1}{\left(\frac{\partial v_{x}}{\partial y}\right)}^{2}dVdt,$$

where *v_x_* is the *x*-direction component of the droplet in the impact process and *V* is the total volume of the droplet, which can be assumed to be constant over the short impact time. It was shown that Δ*E*_v1_ is three orders of magnitude lower than Δ*E*_r1_. This means that the influence of the droplet viscous dissipation could be neglected during the impact process and the subsequent spreading process. What is more, a small part of that is converted into internal kinetic energy in the form of a weak inner vortex flow [41], and the rest is consumed as the droplet spreads further. In this situation, the continuous deformation of the film around the contact line is an elastic ridge that is mainly pushed by the inertia kinetic energy from the droplet.

After reaching the maximum spreading radius, the kinetic energy is exhausted and the evaporation mode is converted to the CCR mode (Figure 4b,c,g,h). Meanwhile, the spreading regime is converted into regime (ii). In this case, the motion of the ridge is mainly transported forward by a lamellar capillary flow that is generated by the film–gas interfacial tension caused by the pseudopartial wetting of the CNT ink droplet [25].

Thus, it was deduced that the different heating temperatures and heating times induced different viscoelastic modulus and surface energy of the PVA film. Furthermore, the different properties of the PVA film significantly influenced the spreading process of the CNT droplet.

**Figure 5:**
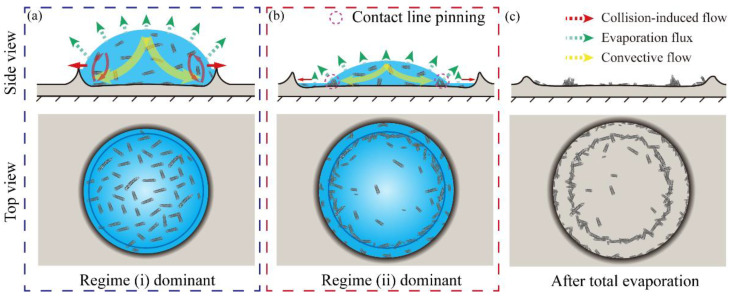
Schematic of the spreading, evaporation, and particle migration process of a single CNT droplet. (**a**) The regime-(i)-dominant spreading. (**b**) The regime-(ii)-dominant spreading. (**c**) After total evaporation and solute migration.

Based on the assumption of contact line pinning, Figure 5 illustrates the migration process of the CNTs during the droplet spreading. The effect of capillary flow generated by rapid evaporation on the droplet’s surface was much stronger than the vortex flow that was produced by the collision, resulting in contact line pinning and the formation of a micro-ring morphology. A small fraction of CNT particles was driven by a lamellar capillary flow that moved along the ridge and finally forming a lighter coffee ring near the inside of the ridge after the ridge stopped moving. This unique migration process formed patterns of double coffee rings on the PVA film.

To further study the effect of the different film properties on the spreading process of the CNT droplet, the characteristic length of droplet spreading in different stages was recorded. The spreading ratio *λ*_1_ represents the degree of droplet spreading for regime (i) on different PVA films. *λ*_1_ can be described by:

(9) $$\lambda_{1}=\frac{D_{s}}{D_{0}}\times 100\%,$$

where *D*_s_ is the balanced spreading diameter of the sessile droplet and *D*_0_ is the initial spreading diameter of the sessile droplet, as shown in Figure 4k,m. The dimensionless number *λ*_2_ is used to represent the ratio of the diameter of the ridge ring to the initial spreading diameter of the sessile droplet, which can be described by:

(10) $$\lambda_{2}=\frac{D_{r}}{D_{0}}\times 100\%,$$

where *D*_r_ is the final diameter of the ridge ring, as shown in Figure 4n.

**Figure 6:**
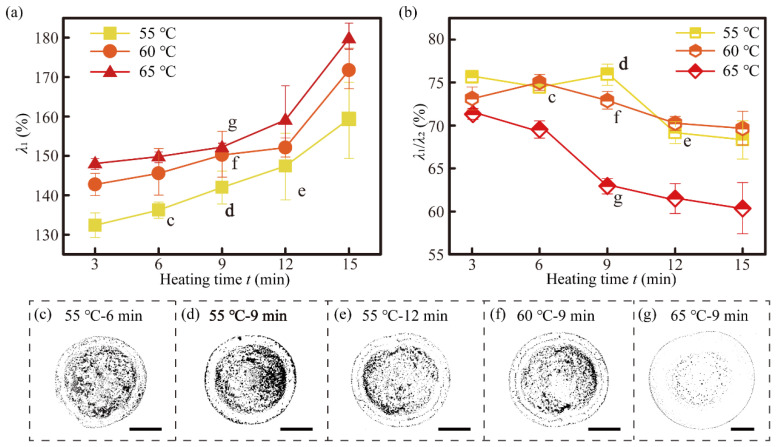
Effects of the films with different heating temperatures and times on the droplet spreading processes and the final morphologies. (**a**) Effect of the heating temperature and time of the films on the droplet spreading ratio *λ*_1_. (**b**) Effect of the pre-processing temperature and time of films on the droplet ring–ridge ratio *λ*_1_/λ_2_. (**c**–**g**) Optical microscope images of the CNT droplet deposition patterns on PVA films that were preprocessed under different heat treatment conditions. All scale bars are 80 μm in (**c**–**g**).

As shown in Figure 6a, *λ*_1_ increased continuously with increased film heating times. *λ*_1_ also increased with the heating temperature increase for the same heating time. In regime (i), the energy loss of the droplet to push the ridge of the film can be expressed as:

(11) $$\Delta E_{r2}=f_{2}\frac{\sigma^{2}}{2G\varepsilon }\int_{t_{i}}^{t_{s}}v(t)D(t)dt,$$

where *f*_2_ is the ratio of the imaginary and real components of the elastic modulus in the spreading process and *t_s_* is the time for droplet spreading to reach equilibrium. Here, the droplets are assumed to have the same kinetic energy before impacting the film. Most dissipated kinetic energy can be counted as Δ*E*_r1_ + Δ*E*_r2_. The viscoelastic modulus of the film decreased with the increase of the heating temperature and the heating time, leading to different spreading degrees of droplets in regime (i).

To further study the differences in the droplet spreading processes on different films in regime (ii), the dimensionless number *λ*_1_/*λ*_2_ was used to represent the extent of the lamella pushing the ridge spreading. As indicated by Figure 6b, *λ*_1_/*λ*_2_ at 55 °C-9 min had the highest value of up to 75.5%. The viscoelasticity decreased with the increase of heating time, while the film–air interfacial tension increased with heating time increasing at the same heating temperature. However, as the heating temperature approached the glass transition temperature, the decrease in the viscoelasticity modulus became less noticeable. At the same time, the interfacial tension increased significantly, resulting in the spreading ratio of the sessile droplets decreasing slightly from 71.2 to 60.3% on the films that were preprocessed at 65 °C. This result can be explained by the large film–air interfacial tension, which dominated the process of spreading and was induced by a long heating time, resulting in the long spreading time in regime (ii).

To reveal the relationship between the droplet spreading process and the final morphologies of the deposited droplets, various micro-ring patterns that were obtained on different PVA films were photographed using an optical microscope. As can be seen from Figure 6c–g, the CNT patterns consisted of two parts: one was the internal circle-like pattern, whose diameter was approximately equal to *D*_s_, and the other was the fine external ring. The fine external ring corresponded to the position of the ridge, while the edge of the inner circle-like pattern corresponded to the position where the CCR evaporation mode began to emerge. The pattern with the minimum spacing between the inner pattern and the outer ring at maximum *λ*_2_ (Figure 6d) was conducive for the further printing of low-porosity CNT patterns.

With the same jetting velocity and initial drop size, the favourable CNT pattern corresponding to the maximum *λ*_2_, *t* = 9 min was obtained for the PVA film preprocessed at 55 °C. Therefore, in the following experiments, CNT lines and patterns were printed on films preprocessed at 55 °C for 9 min.

### 3.3. Effect of the Number of Staggered Overwriting Layers on the Line Morphologies of CNTs under Constant Spacing

As essential components of CNT-based conductive patterns, compact and uniform CNT lines should be printed by elaborately adjusting the deposition parameters, including the droplet spacing ratio *δ*, the jetting frequency *f*, and the number of overwriting layers *N*. The droplet spacing ratio *δ* is defined as:

(12) $$\delta =\frac{d_{1}}{D_{s}}\times 100\%,$$

where *d*_1_ is the droplet spacing, as shown in Figure 6a.

According to Ihnen [21] and Lian [30], the inkjet-printed lines easily have bulging and discontinuous defects that are caused by the coalescence between sequentially deposited droplets. Such coalescence is due to the longitudinal inner flow that is induced by Laplace pressure. To avoid the bulging defect, we printed stable single-layer CNT lines in the total-drying regime [30] by choosing a *δ* of 50% and an ejection frequency of 2 Hz. In the experiment, the stage velocity *v*_s_ was determined using:

(13) $$v_{s}=d_{1}/\frac{1}{f}$$

However, the distribution of CNTs inside the monolayer pattern was non-uniform. Hole defects could be observed all over the printed patterns, indicating an adverse effect on the performance of CNT-based devices. To obtain high-quality CNT patterns, uniform and dense CNT lines need to be printed first. Here, we proposed a staggered overwriting strategy to print hole-free CNT lines on the PVA film, as shown in Figure 6a–c. The *y*-axis position of the initial droplet in the second layer was the same as that in the first layer. The offset printing step in the printing direction (*x*-axis) is *d*_2_.

**Figure 7:**
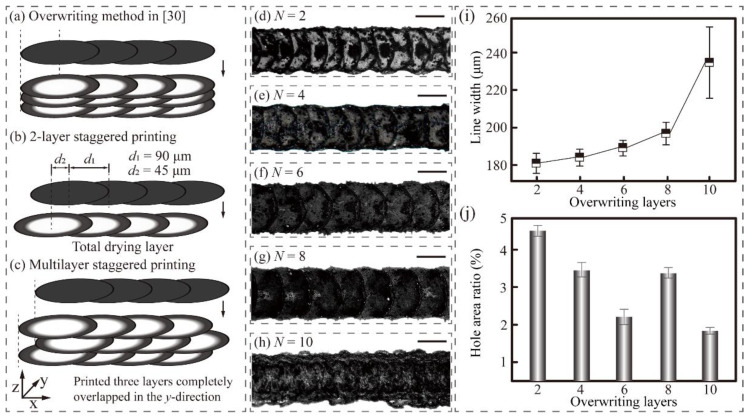
Effect of overwriting layers on the morphologies of printed CNT lines. (**a**–**c**) Schematic illustration of CNT line printing and staggered overwriting printing. (**d**–**h**) Optical microscope images of printed CNT lines with different numbers of layers (2 to 10 layers). All CNT lines were printed within 1 min after each PVA film’s heat treatment. All scale bars are 110 μm. (**i**) Effect of the number of layers on the line width. (**j**) Effect of the number of layers on the ratio of the hole area to the line area.

As shown in Figure 7d–j, when the number of layer *N* increased from 2 to 10, the line width *w_L_* continuously increased from 180.5 to 246.5 μm. The reason lies in that the viscous energy dissipation decreased as the droplets spread over the film, resulting in an increase in pinning radius. Note that the line width suddenly increased with an amplitude of 46.5 μm when *N* increased from 8 to 10. This change might have been caused by the weakened pinning of the upper droplets, inducing some of the CNT solute to pass over the ridge, resulting in uneven protrusions at the edge. As *N* increased from 2 to 6, the porosity in the overwriting lines significantly decreased from 4.9 to 2.2% (Figure 7j). The kernel, which was formed by CNT particle aggregation in the line centre, decreased as the CNT particles were diffused to fill hole defects. When the layer number increased to 8, new hole defects could be formed again in the original kernel area due to the further diffusion of CNT particles. When *N* reached 10, these hole defects were covered again by the CNT solute, resulting in a renewed decrease in the porosity. Finally, we overwrote six layers of CNT lines on 55 °C-9 min PVA films using a staggered strategy to obtain uniform and dense CNT lines with a resolution of 190.5 μm.

**Figure 8:**
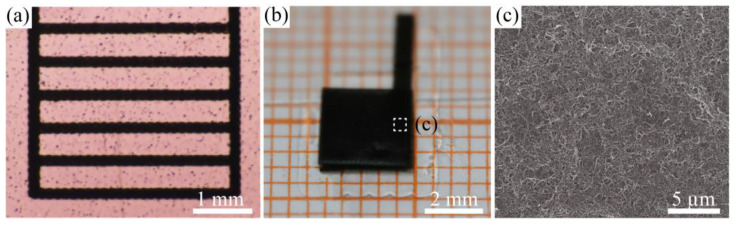
Optical microscope images of a printed (**a**) micro-ribbon and (**b**) patterned PVA-CNT thin film. (**c**) SEM image of the printed CNT film on the preprocessed PVA film. All patterns were obtained by overwriting 6 layers of CNT lines on 55 °C-9 min PVA films in the aforementioned staggered strategy.

After the elaborate investigation on multi-layer overwriting, the micro-ribbon circuits were printed on the PVA film with favourable outlines, as shown in Figure 8a. Figure 8b shows a rectangular PVA-CNT thin film with a footprint that was printed using the integrated fabrication method. The top-down SEM image of the PVA-CNT film, which had no obvious holes, is shown in Figure 8c. These printed patterned electronics demonstrate the feasibility of the integrated fabrication method for PVA-CNT electronics regarding flexibility and reliability.

## 4. Conclusions

In this study, an effective method for the integrated fabrication of patterned PVA films of micron thickness and uniform CNT micropatterns on the surface of a preprocessed PVA film was first proposed. The effects of writing parameters on the thickness of PVA film, the effects of different heat treatment parameters of PVA film on the morphology of CNT droplets after spreading, and the effect of overwriting layers on the printing morphology of straight CNT lines on the preprocessed PVA film were experimentally and theoretically investigated.

Patterned PVA films of 1–5 μm thickness could be controllably written by regulating the writing gap, the substrate velocity, and the working pressure. The thickness of the written PVA films was in good agreement with the curve of theoretical models, where the R^2^ of all parameters was found to be greater than 0.97. The written 5 μm thickness PVA films were heated at different heating temperatures for different heating times to obtain a smooth surface and different properties (i.e., the viscoelastic modulus and the surface energy). After that, the spreading processes of the CNT–isopropanol sessile droplets on different viscoelastic thin PVA films were studied. We have observed that the droplet-spreading process exhibits two regimes that pushed the ridge on the film to move. Before the equilibrium contact diameter was reached, the ridge was mainly pushed by the droplet’s initial kinetic energy. After reaching the equilibrium contact diameter, the droplet was pinned due to the accumulation of CNT particles and the ridge was mainly transported forward by a lamellar capillary flow that was generated by the film–gas interfacial tension that was caused by the droplet’s pseudopartial wetting. This mechanism explains why the minimum ring–ridge ratio was produced on the PVA film heated at 55 °C for 9 min. Then, the uniform and compact CNT lines with favourable outlines and low porosity of 2.2% were overwritten for six layers on the 55 °C-9 min PVA film with a staggered overwriting strategy. Finally, a patterned PVA-CNT film and micro-ribbon were printed using this hybrid printing method.

This study opens new routes for the integrated fabrication of micron-thickness PVA-CNT electronics that consist of a patterned PVA film and CNT micropatterns. This work can contribute to the morphology regulation of CNT droplet printing on a thin viscoelastic film and the fabrication of straight and compact lines to apply CNT-based functional devices. Three-dimensional integrated micro-supercapacitors were printed using this method [11]. The regulation strategy and characterisation method of the morphology generated by the nanosuspension droplet impacting and spreading over the thin viscoelastic film will be helpful for the printing of micron-thickness carbon-based electronics of other materials.

## Data Availability

The data presented in this study are available on request from the corresponding author.

## Supplementary Materials

The following are available online at https://www.mdpi.com/article/10.3390/nano11092335/s1, Figure S1. (a) Schematic diagram of the equipment of the micro-pen-writing technology. (b) Local amplification diagram of micro-pen writing PVA polymer; Figure S2. (a) Diagram of the equipment of the self-development drop-on-demand equipment. (b) Partial enlarged picture of the DOD print-head. (c) Partial enlarged picture of the nozzle

## Appendix A

First of all, the impact dynamics is characterised by the Weber and Reynolds numbers, which are defined using the liquid properties as:

(A1) $$\mathrm{We}=\frac{\rho_{1}v^{2}d_{\mathrm{drop}}}{\sigma },$$

and

(A2) Re=ρlddropvμ1,

where *ρ*_1_, *v*, *σ*, and *μ*_1_ are the density (820 kg/m^3^), falling velocity (2 m/s), surface tension (2.2 × 10^−2^ N/m), and dynamic viscosity (2.1 × 10^−3^ Pa·s) of the CNT droplets, respectively. The dimensionless thickness of the film *h*^*^:

(A3) $$h^{*}=h/d_{\mathrm{drop}},$$

was used to measure the size of PVA film relative to droplet diameter. We, Re, and *h*^*^ were calculated to be 20.127, 105.428, and 0.037, respectively. According to Wang [42], the impact target film can be seen as a thin film when *h*^*^ < 0.1. Weiss [43] concluded that the droplet only spreads over the liquid film surface and forms capillary waves propagating around when We >> 40. As stated above, droplets only spread without splashing after impacting the PVA film. The roughness of the glass slide as the rigid support was 0.006 μm, which was far less than the film thickness, whose influence on the droplets could be neglected [44]. The Froude number of droplets can be expressed as:

(A4) $$\mathrm{Fr}=\frac{v^{2}}{gd_{\mathrm{drop}}},$$

where *g* is the acceleration of gravity, which is approximately equal to 9.8 m/s^2^. Here, Fr is equal to 2148.228, meaning that the effect of the droplet’s gravity on the dynamics can be neglected [41].

## References

[1] Ji H., Zhao X., Qiao Z., Jung J., Zhu Y., Lu Y., Zhang L., MacDonald A., Ruoff R.S. (2014). Capacitance of carbon-based electrical double-layer capacitors. Nat. Commun..

[2] Pei Z., Hu H., Liang G., Ye C. (2017). Carbon-Based Flexible and All-Solid-State Micro-supercapacitors Fabricated by Inkjet Printing with Enhanced Performance. Nano-Micro Lett..

[3] Tai Y., Lubineau G. (2017). Human-Finger Electronics Based on Opposing Humidity-Resistance Responses in Carbon Nanofilms. Small.

[4] Jang S., Kim B., Geier M.L., Hersam M.C., Dodabalapur A. (2015). Short Channel Field-Effect-Transistors with Inkjet-Printed Semiconducting Carbon Nanotubes. Small.

[5] Lin Z., Le T., Song X., Yao Y., Li Z., Moon K.-S., Tentzeris M.M., Wong C.-P. (2013). Preparation of Water-Based Carbon Nanotube Inks and Application in the Inkjet Printing of Carbon Nanotube Gas Sensors. J. Electron. Packag..

[6] Mass M., Veiga L., Garate O., Longinotti G., Moya A., Ramón E., Villa R., Ybarra G., Gabriel G. (2021). Fully Inkjet-Printed Biosensors Fabricated with a Highly Stable Ink Based on Carbon Nanotubes and Enzyme-Functionalized Nanoparticles. Nanomaterials.

[7] Huang X., Xie R., Sugime H., Noda S. (2021). Performance enhancement of carbon nanotube/silicon solar cell by solution processable MoO. Appl. Surf. Sci..

[8] Yu Y., Chen S., Jia Y., Qi T., Xiao L., Cui X., Zhuang D., Wei J. (2020). Ultra-black and self-cleaning all carbon nanotube hybrid films for efficient water desalination and purification. Carbon.

[9] Kumar R., Khan M.A., Anupama A., Krupanidhi S.B., Sahoo B. (2021). Infrared photodetectors based on multiwalled carbon nanotubes: Insights into the effect of nitrogen doping. Appl. Surf. Sci..

[10] Mitin D., Berdnikov Y., Vorobyev A., Mozharov A., Raudik S., Koval O., Neplokh V., Moiseev E., Ilatovskii D., Nasibulin A.G. (2020). Optimization of Optoelectronic Properties of Patterned Single-Walled Carbon Nanotube Films. ACS Appl. Mater. Interfaces.

[11] Hu J., Luo J., Xu Z., Xie K., Yu H., Wang H., Shen C., Qi L.-H., Wei B. (2021). Hybrid printed three-dimensionally integrated micro-supercapacitors for compact on-chip application. Appl. Phys. Rev..

[12] Yu W., Zhou H., Li B.Q., Ding S. (2017). 3D Printing of Carbon Nanotubes-Based Microsupercapacitors. ACS Appl. Mater. Interfaces.

[13] Park S., Heo S.W., Lee W., Inoue D., Jiang Z., Yu K., Jinno H., Hashizume D., Sekino M., Yokota T. (2018). Self-powered ultra-flexible electronics via nano-grating-patterned organic photovoltaics. Nat. Cell Biol..

[14] Reinheimer T., Baumann V., Binder J.R. (2020). Fabrication of Flexible Multilayer Composite Capacitors Using Inkjet Printing. Nanomaterials.

[15] Lan W., Chen Y., Yang Z., Han W., Zhou J., Zhang Y., Wang J., Tang G., Wei Y., Dou W. (2017). Ultraflexible Transparent Film Heater Made of Ag Nanowire/PVA Composite for Rapid-Response Thermotherapy Pads. ACS Appl. Mater. Interfaces.

[16] Asuo I.M., Fourmont P., Ka I., Gedamu D., Bouzidi S., Pignolet A., Nechache R., Cloutier S.G. (2018). Highly Efficient and Ultrasensitive Large-Area Flexible Photodetector Based on Perovskite Nanowires. Small.

[17] Zhang C., Fan Y., Li H., Li Y., Zhang L., Cao S., Kuang S., Zhao Y., Chen A., Zhu G. (2018). Fully Rollable Lead-Free Poly(vinylidene fluoride)-Niobate-Based Nanogenerator with Ultra-Flexible Nano-Network Electrodes. ACS Nano.

[18] Yun T.G., Park M., Kim D.-H., Kim D., Cheong J.Y., Bae J.G., Han S.M., Kim I.-D. (2019). All-Transparent Stretchable Electrochromic Supercapacitor Wearable Patch Device. ACS Nano.

[19] Hu W., Huang W., Yang S., Wang X., Jiang Z., Zhu X., Zhou H., Liu H., Zhang Q., Zhuang X. (2017). High-Performance Flexible Photodetectors based on High-Quality Perovskite Thin Films by a Vapor-Solution Method. Adv. Mater..

[20] Lee K.-H., Lee S.-S., Ahn D.B., Lee J., Byun D., Lee S.-Y. (2020). Ultrahigh areal number density solid-state on-chip microsupercapacitors via electrohydrodynamic jet printing. Sci. Adv..

[21] Ihnen A.C., Petrock A.M., Chou T., Fuchs B.E., Lee W.Y. (2012). Organic Nanocomposite Structure Tailored by Controlling Droplet Coalescence during Inkjet Printing. ACS Appl. Mater. Interfaces.

[22] Homenick C.M., James R., Lopinski G.P., Dunford J., Sun J., Park H., Jung Y., Cho G., Malenfant P.R.L. (2016). Fully Printed and Encapsulated SWCNT-Based Thin Film Transistors via a Combination of R2R Gravure and Inkjet Printing. ACS Appl. Mater. Interfaces.

[23] He P., Derby B. (2017). Inkjet printing ultra-large graphene oxide flakes. 2D Mater..

[24] Soum V., Park S., Brilian A.I., Kim Y., Ryu M.Y., Brazell T., Burpo F.J., Parker K.K., Kwon O.-S., Shin K. (2019). Inkjet-Printed Carbon Nanotubes for Fabricating a Spoof Fingerprint on Paper. ACS Omega.

[25] Sebilleau J. (2013). Equilibrium Thickness of Large Liquid Lenses Spreading over Another Liquid Surface. Langmuir.

[26] Carré A., Gastel J.-C., Shanahan M.E.R. (1996). Viscoelastic effects in the spreading of liquids. Nat. Cell Biol..

[27] Hondred J.A., Stromberg L., Mosher C.L., Claussen J.C. (2017). High-Resolution Graphene Films for Electrochemical Sensing via Inkjet Maskless Lithography. ACS Nano.

[28] Deegan R.D., Bakajin O., Dupont T.F., Huber G., Nagel S.R., Witten T.A. (1997). Capillary flow as the cause of ring stains from dried liquid drops. Nature.

[29] Shin K.-Y., Hong J.-Y., Jang J. (2011). Micropatterning of Graphene Sheets by Inkjet Printing and Its Wideband Dipole-Antenna Application. Adv. Mater..

[30] Lian H., Qi L., Luo J., Hu K. (2019). Uniform nitrogen-doped graphene lines with favorable outlines printed by elaborate regulation of drying and overlapping. Appl. Surf. Sci..

[31] Chen Y., Askounis A., Koutsos V., Valluri P., Takata Y., Wilson S.K., Sefiane K. (2020). On the Effect of Substrate Viscoelasticity on the Evaporation Kinetics and Deposition Patterns of Nanosuspension Drops. Langmuir.

[32] Iqbal R., Matsumoto A., Sudeepthi A., Shen A.Q., Sen A.K. (2019). Substrate stiffness affects particle distribution pattern in a drying suspension droplet. Appl. Phys. Lett..

[33] Karpitschka S., Pandey A., Lubbers L., Weijs J.H., Botto L., Das S., Andreotti B., Snoeijer J.H. (2016). Liquid drops attract or repel by the inverted Cheerios effect. Proc. Natl. Acad. Sci. USA.

[34] Lian H., Qi L., Luo J., Zhang R. (2021). Drop-on-demand printing of edge-enhanced and conductive graphene twin-lines by coalescence regulation and multi-layers overwriting. 2D Mater..

[35] Zhou L. (2008). Study on Fabrication of Microbridge Structure by Micropen Direct Writing Technique. Master’s Thesis.

[36] Kajiya T., Daerr A., Narita T., Royon L., Lequeux F., Limat L. (2013). Advancing liquid contact line on visco-elastic gel substrates: Stick-slip vs. continuous motions. Soft Matter.

[37] Eggers J., Fontelos M.A., Josserand C., Zaleski S. (2010). Drop dynamics after impact on a solid wall: Theory and simulations. Phys. Fluids.

[38] Karpitschka S., Das S., Van Gorcum M., Perrin H., Andreotti B., Snoeijer J.H. (2015). Droplets move over viscoelastic substrates by surfing a ridge. Nat. Commun..

[39] Ersoy N.E., Eslamian M. (2019). Capillary surface wave formation and mixing of miscible liquids during droplet impact onto a liquid film. Phys. Fluids.

[40] Alizadeh A., Bahadur V., Shang W., Zhu Y., Buckley D., Dhinojwala A., Sohal M. (2013). Influence of Substrate Elasticity on Droplet Impact Dynamics. Langmuir.

[41] Clanet C., Béguin C., Richard D., Quéré D. (2004). Maximal deformation of an impacting drop. J. Fluid Mech..

[42] Wang A.-B., Chen C.-C. (2000). Splashing impact of a single drop onto very thin liquid films. Phys. Fluids.

[43] Weiss D.A., Yarin A.L. (1999). Single drop impact onto liquid films: Neck distortion, jetting, tiny bubble entrainment, and crown formation. J. Fluid Mech..

[44] Yarin A.L., Weiss D.A. (1995). Impact of drops on solid surfaces: Self-similar capillary waves, and splashing as a new type of kinematic discontinuity. J. Fluid Mech..

