# Supplementary material for: Direct Fabrication of Micron-Thickness PVA-CNT Patterned Films by Integrating Micro-Pen Writing of PVA Films and Drop-on-Demand Printing of CNT Micropatterns
Source: Nanomaterials (Basel). 2021 Sep 8;11(9):2335. doi: 10.3390/nano11092335 (PMC8466232; doi:10.3390/nano11092335)
Supplement: Supplementary file 1 [file nanomaterials-11-02335-s001.zip › nanomaterials-1365938-supplementary.pdf]

Supplementary material

Direct Fabrication of Micron-Thickness PVA-CNT Patterned Films by Integrating Micro-Pen Writing of PVA Films and Drop-on-Demand Printing of CNT Micropatterns

Jun Luo [1,2,*], Zhixuan Zhao [1,2], Lehua Qi [1,2,*], Hongcheng Lian [1,2] and Yufang Zhao [1]

1 School of Mechanical Engineering, Northwestern Polytechnical University, Xi'an 710072, China; zhaozhixuan97@mail.nwpu.edu.cn (Z.Z.); 2015100416@mail.nwpu.edu.cn (H.L.); 2020100897@mail.nwpu.edu.cn (Y.Z.)
2 Research & Development Institute of Northwestern Polytechnical University in Shenzhen, School of Mechanical Engineering, Northwestern Polytechnical University, Xi'an 710072, China
* Correspondence: luojun@nwpu.edu.cn (J.L.); qilehua@nwpu.edu.cn (L.Q.)

As shown in Figure.S1 (a), the equipment of micro-pen-writing consists of a picpliterpump connecting a pressure valve for controling writing pressure, a micro-pen including a storage chamber and a nozzle, an industrial personal computer (IPC) and 3D-piezo-nano-stages. By applying a certain pressure using picpliterpump, the PVA solution in the storage chamber is extruded out of thenozzle. Under the control of the IPC, the PVA solution is directly writen on the substrate, which moves along the setting path, so as to obtain the pre-designed pattern and complete the purpose of direct writing of micro-pen-writing.

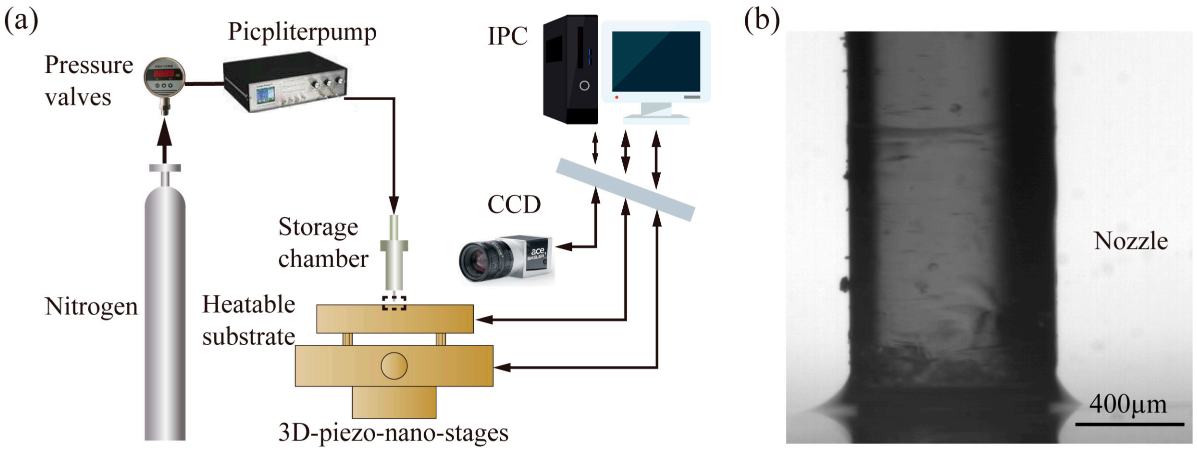

Figure S1. (a) Schematic diagram of the equipment of the micro-pen-writing technology. (b) Local amplification diagram of micro-pen writing PVA polymer.

The droplet printing equipment was composed of a DOD (drop-on-demand) print-head driven by a piezoelectric ceramic tube, a signal driver (Model 9214, Quantum Composers, US) for the pulse voltage generation with a signal amplifier.

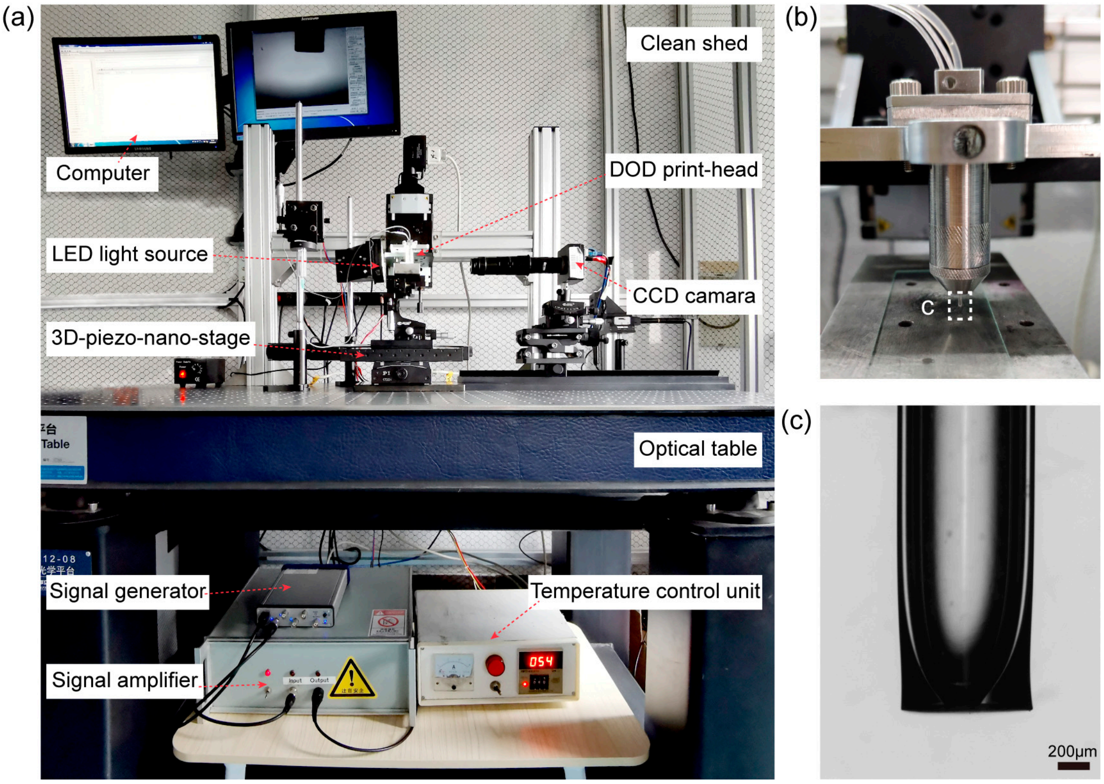

Figure S2. (a) Diagram of the equipment of the self-development drop-on-demand equipment. (b) Partial enlarged picture of the DOD print-head. (c) Partial enlarged picture of the nozzle.
